# Visual detection and differentiation of porcine epidemic diarrhea virus wild−type strains and attenuated vaccine strains using CRISPR/Cas13a-based lateral flow strip

**DOI:** 10.3389/fcimb.2022.976137

**Published:** 2022-09-13

**Authors:** Dongdong Yin, Lei Yin, Hao Guo, Jieru Wang, Xuehuai Shen, Ruihong Zhao, Xiaocheng Pan, Yin Dai

**Affiliations:** ^1^ Institute of Animal Husbandry and Veterinary Science, Anhui Academy of Agricultural Sciences, Livestock and Poultry Epidemic Diseases Research Center of Anhui Province, Anhui Province Key Laboratory of Livestock and Poultry Product Safety Engineering, Hefei, China; ^2^ Animal Health Supervision Institute, Feixi County Agricultural and Rural Bureau, Hefei, China

**Keywords:** PEDV wild-type strain, attenuated vaccine strain, CRISPR/Cas13a, RAA, lateral flow detection

## Abstract

Porcine epidemic diarrhea virus (PEDV) is an enteric coronavirus that causes acute watery diarrhea and vomiting in unweaned piglets. Infections result in high mortality and serious economic losses to the swine industry. PEDV attenuated vaccine does not completely protect against all mutant wild-type strains, and PEDV infection can periodically occur. A sensitive, accurate, and simple detection method for PEDV is needed to reduce the occurrence of the disease. In this study, the CRISPR/Cas13a system was combined with recombinase aided amplification to develop a rapid diagnostic method to distinguish PEDV wild-type strains from attenuated vaccine strains. The method is based on isothermal detection at 37°C. The results are used for visual readout. The assay had high sensitivity and specificity, with a detection limit of 10^1^ copies/μL for the gene of interest, and no cross-reactivity with other pathogens. The Cas13a detection worked well with clinical samples. This visual, sensitive, and specific nucleic acid detection method based on CRISPR/Cas13a should be a powerful tool for detecting PEDV.

## Introduction

Porcine epidemic diarrhea (PED) is an acute, highly contagious enteric infectious disease caused by porcine epidemic diarrhea virus (PEDV). Diarrhea, vomiting, and dehydration are the main symptoms in piglets. PED was first reported in the United Kingdom in 1971 and was significantly more lethal in suckling pigs than other age groups (Song and Park, 2012; Chen et al., 2014; Jung and Saif, 2015). In 2006, the appearance of PEDV in immunized pigs was described, indicating that the immunization effect of PEDV vaccine was not ideal (Li et al., 2012). After 2010, PEDV mutant strains appeared in China and spread rapidly across the country (Sun et al., 2012; Lin et al., 2014; Sun et al., 2015). To combat the variant of PEDV, new commercial vaccines based on the classic PEDV strain ZJ08 and the variant strain AJ1102 have been developed in China in recent years. These vaccines have achieved a relative control of PED and have alleviated disease progression (Wang et al., 2016; Li et al., 2017). Therefore, establishing a diagnostic method that can distinguish between PEDV wild-type and vaccine strains, and can exclude the influence of other pathogens, will help reduce the possibility of immunized pigs being culled and reduce economic losses.

At present, viral isolation, quantitative real-time PCR (qRT-PCR), loop-mediated isothermal amplification, and enzyme linked immunosorbent assay are usually used to test whether pigs are infected with PEDV (Yu et al., 2015; Valko et al., 2019; Wang et al., 2020b). These methods are complicated to operate, require special instruments, or have high false positives and are difficult to use for rapid and accurate on-site testing. Therefore, a new technical solution is required. CRISPR/Cas13a can activate its collateral cleavage activity after cleaving target RNA under the guidance of specific crispr RNA (crRNA) and cleave surrounding non-specific RNA (Abudayyeh et al., 2017; Myhrvold et al., 2018; Wang et al., 2020a). Based on this principle, nonspecific RNA reporter probes can be added to the reaction system to achieve specific detection of target molecules. They can be combined with recombinase aided amplification (RAA) with CRISPR/Cas13a detection to establish specific high sensitivity enzymatic reporter unlocking (SHERLOCK) (Kellner et al., 2019). The method is convenient, rapid, sensitive, and specific. The approach also provides a new avenue for the development of pathogen nucleic acid detection technology. CRISPR/cas13a-based assays have been applied to detect porcine reproductive and respiratory syndrome virus (PRRSV), Avian influenza A (H7N9) virus, Ebola virus, dengue virus, Zika virus, and Tembusu virus (Liu et al., 2019b; Qin et al., 2019; Chang et al., 2020; Yin et al., 2022). To construct a more convenient method to detect and distinguish PEDV attenuated vaccine strains from wild-type virus strains, in the present study we developed a detection system by combining CRISPR/Cas13a, RAA, and lateral flow strips.

The open reading frame 3 (ORF3) gene of PEDV is one of the determinants of PEDV virulence. Attenuated strains generally delete dozens of bases in the ORF3 gene. Sequences around the attenuated deletion region are relatively conserved, which provides a good basis for the identification of PEDV wild-type and attenuated vaccine strains (Li et al., 2014; Su et al., 2016; Lu et al., 2020). We designed specific primers and crRNAs for the deletion region of ORF3 gene and tested their specificity using clinical samples. We believe the method can be an alternative to field PEDV detection and reduce livestock losses by enabling early control of the spread of PEDV wild-type strains.

## Materials and methods

### Viruses and clinical samples

PEDV attenuated vaccine strain CV777, PEDV wild strain AH2021, porcine circovirus type 2 (PCV-2), and porcine circovirus type 3 (PCV3) were maintained in our laboratory. PRRSV, Pseudorabies virus (PRV), and classical swine fever virus (CSFV) vaccine was produced by Wuhan Keqian Biology Co., Ltd. Forty-two fecal and intestinal tissue samples were collected from farms located in Anhui Province that housed pigs with diarrhea.

### RAA primer design and crRNA preparation

**Figure 1:**
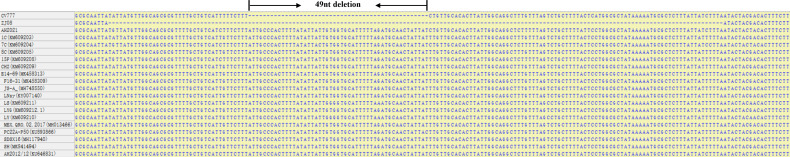
*ORF3* regions alignment results of PEDV wild-types strains and attenuated vaccine strains CV777.

Compared with the ORF3 genes of attenuated vaccine strain and different PEDV wild-type whose sequences were available in GenBank (**Figure 1**), we found that the attenuated vaccine strain CV777 has a 49-nucleotide deletion sequence compared with the wild-type strains. The RAA primers were selected in the deleted segment from the ORF3 gene alignment between the attenuated vaccine strain and the wild-type strain. The T7 promoter sequence (GAAATTAATACGACTCACTATAGGG) was appended to the 5’ end of the RAA forward primer. A target sequence of 28 bp after the adjacent motif sequence was designed according to the website CRISPR-DT (https://cas13design.nygenome.org/) for the RAA-amplified products of ORF3 gene, and a stem-loop structure sequence (GATTTAGACTACCCCAAAAACGAAGGGGACAAAAC) added to the 5’ of the target sequence to form crRNA-F, and the reverse complement was crRNA-R. For crRNA preparation, the DNA templates of crRNA were appended with the T7 promoter sequence and synthesized as primers by General Biological System (Anhui) Co. (**Table 1**). The lateral-flow-reporter (5’-/56-FAM/mArArUrGrGrCmAmArArUrGrGrCmA/3Bio/-3’) was synthesized by General Biological System (Anhui). Two oligonucleotides were annealed to a double‐stranded DNA using Annealing Buffer for DNA Oligos (Beyotime, China). The double-stranded DNA was purified by gel extraction. According to the instructions of the HiScribe T7 Quick High Yield RNA Synthesis kit (NEB, USA), the double-stranded DNA was transcribed to crRNA. Finally, crRNA was purified using NucAway™ Spin Columns (Invitrogen, USA) according to the manufacturer’s instructions and stored at −80°C.

**Table:** Table 1 The crRNA, primers, and probes used in this study.

Primers	Sequences (5’-3’)
ORF3-F ORF3-R	TGGACTTTTTCAATACACGATTG CATTCACTAATTGTAGCATACTCG
RAA-F RAA-R	GAAATTAATACGACTCACTATAGGGTTCCAATTAGACAAGCTTCAAATGTGACGGG CGCCAGGAGTAAAAGCAGACTAAACAAAGCC
qRT-PCR-F qRT-PCR-R	ATTGCCCACTTTTATATTATTGTGG TGCCGCCCACGTATAGCTAGATACA
crRNA1-F	GATTTAGACTACCCCAAAAACGAAGGGGACAAAACATCCATGATCCAGTAGGGGGTCTGCGTG
crRNA1-R	CACGCAGACCCCCTACTGGATCATGGATGTTTTGTCCCCTTCGTTTTTGGGGTAGTCTAAATC
crRNA2-F	GATTTAGACTACCCCAAAAACGAAGGGGACTAAAACCCACGTTCATGCCCGAACGCTCCGAGTT
crRNA2-R	AACTGGCCGTACCCGCTCTGCGGTCAGCGTTTTAGTCCCCTTCGTTTTTGGGGTAGTCTAAATC

### Nucleic acid preparation

The viral genomic nucleic acids of the PEDV attenuated vaccine strain CV777 and wild-type strain AH2021, PCV2, PCV3, PRRSV, PRV, and CSFV were extracted with a TIANamp Virus DNA/RNA Kit (Tiangen, China) according to the manufacturer’s instructions and stored at -80°C until use. PEDV isolate AH2012/12 (GenBank accession KU646831) was donated by researcher Li Bin of Jiangsu Academy of Agricultural Sciences.

### Cas13a nucleic acid detection

RAA reactions were conducted using the RAA kit according to the manufacturer’s instructions (Magiltd, China). One µL of cDNA or DNA was amplified in a 50-μL reaction system for 30 min at 37°C. The RAA reaction products were transferred to the CRISPR/Cas13a cleavage assay. CRISPR/Cas13a lateral flow detection reaction system was performed with 45 nM LwCas13a (Magiltd, China), 22.5 nM crRNA, 125 nM lateral-flow-reporter, 0.25 µL RNase inhibitor, 1 mM dNTP, 0.4 µL T7 RNA polymerase (NEB, USA), and 1 µL RAA reaction products. The RAA reaction products were replaced with a nuclease-free water sample as a negative control. The reactions were performed at 37°C for 40 min. Ten µL of the detection products were transferred into 90 µL detection buffer (Magiltd, China), loaded onto lateral flow strip, and left for 5 min to observe results.

### Specificity and sensitivity of the Cas13a nucleic acid detection

ORF3 gene fragments (ranging from 24,799–25,461 bp of AH2012/1) were cloned and inserted into the pMD-19T vector, named pMD-19T-ORF3, to create a plasmid DNA standard. Aliquots of pMD19T-ORF3 ranging from 1.0 × 108 to 1.0 × 100 copies/μL were prepared as a template for Cas13a lateral flow detection. The specificity of lateral flow detection was assessed using the genomic cDNA or DNA of a panel of pathogens, including PCV2, PCV3, PRRSV, PRV, and CSFV.

### Validation with clinical samples

A total of 42 fecal and intestinal tissue samples were collected from six pig farms in Anhui Province, six of these were from piglets that were inoculated with the attenuated vaccine CV777, and another 36 fecal and intestinal tissue samples were collected from piglets with symptoms of diarrhea. All samples were used to confirm the applicability of PEDV-specific lateral flow in clinical diagnosis. The results were compared with those obtained with qRT-PCR described previously, which was run in parallel for the above clinical samples (Yang et al., 2021).

## Results

### Establishment of CRISPR/Cas13a lateral flow detection

**Figure 2:**
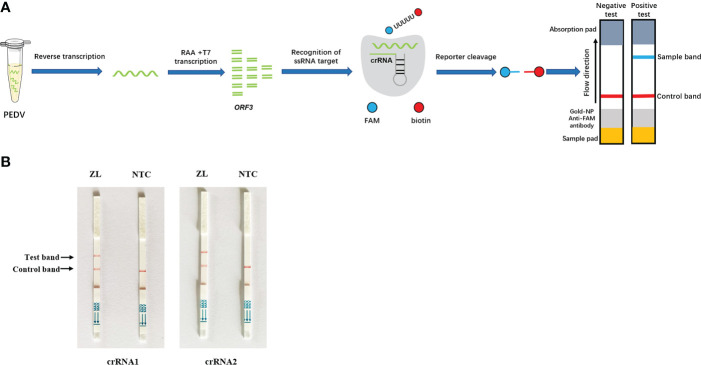
Establishment of the CRISPR/Cas13a lateral flow detection. **(A)** Schematic illustration of the workflow for Cas13a PEDV testing. The ORF3 gene in the PEDV genome was amplified by RAA and transcribed into RNA that was recognized by crRNA and activated Cas13a nuclease. The activated Cas13a nuclease then cleaved the reporter molecule, and the reporter molecule could appear as a band on the test strip. **(B)** Analysis of different crRNAs by CRISPR/Cas13a lateral flow detection. ZL, pMD19T-ORF3 standard plasmid and NTC, negative control.

For the on-site detection, we performed RAA combined with CRISPR/Cas13a to detect PEDV according to the schematic diagram in **Figure 2A**. Lateral flow assays used the FAM-RNA-biotin reporter. For the positive samples, the FAM-RNA-biotin reporter was cleaved to aggregate gold-nanoparticle (NP)-anti-FAM antibody conjugates at the test band and reduce aggregation at the control band. For the negative sample, the gold-NP-anti-FAM antibody was fully conjugated to the FAM-RNA-biotin reporter and the conjugate was captured by the biotin ligand in the control band. Two Cas13a crRNAs targeting the RAA-amplified products of PEDV ORF3 were designed. To verify the effectiveness of the designed crRNAs, we performed Cas13a experiments using the RAA products as templates. The experimental groups showed that the combination of primer1+crRNA1 and primer2+crRNA2 could produce obvious positive bands on the lateral flow detection strips (**Figure 2B**).

### Sensitivity of Cas13a lateral flow detection

**Figure 3:**
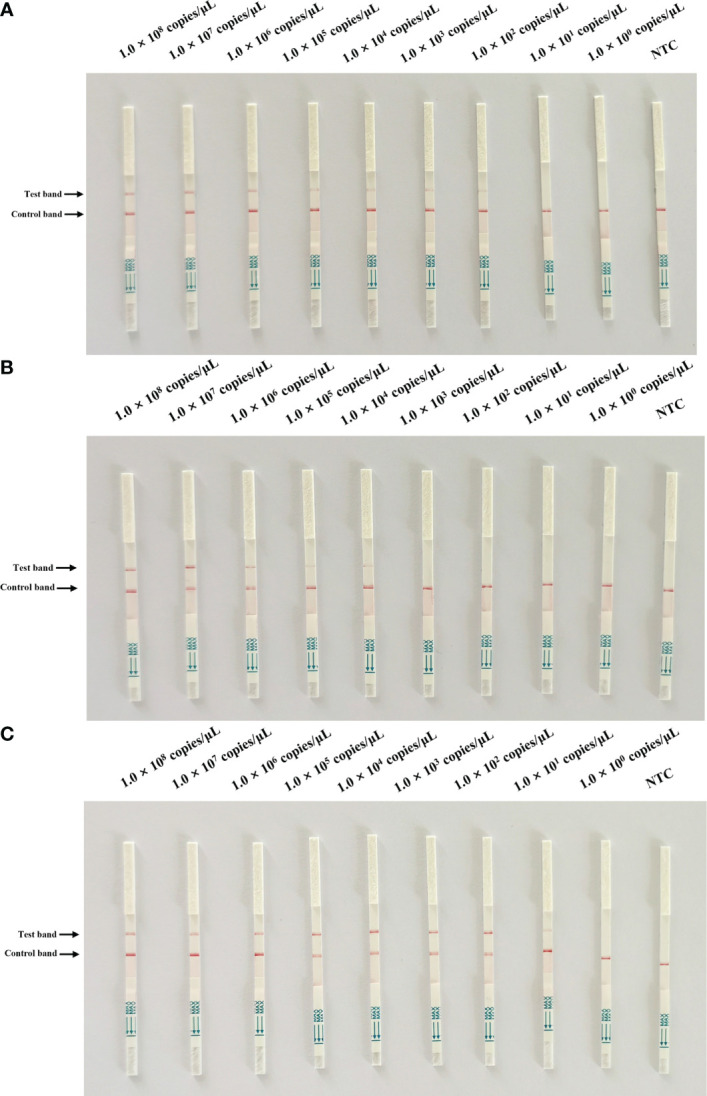
Sensitivity test of Cas13a lateral flow detection. A 10-fold serial dilution of pMD19T-ORF3 was used as the detection template. **(A)** Sensitivity test of the primer1 + crRNA1 combinations. **(B)** Sensitivity test of the primer2 + crRNA2 combinations. **(C)** Sensitivity of the primer2 + crRNA2 combination after reaction system optimization. NTC, negative control.

To optimize the performance of the assay, the sensitivity of the Cas13a lateral flow detection based on different primer pairs and crRNA combinations was determined with 1.0 × 108 to 1.0 × 100 copies of pMD-19T-ORF3 serving as the template. The combination of primer2 + crRNA2 had the highest sensitivity, of which the detection limit could reach 102 copies/µL (**Figure 3A**). The combination of primer1 + crRNA1 had poor sensitivity, and the lower detection limit could only reach 104 copies/µL (**Figure 3B**). To further improve the sensitivity of this assay, the primer2 + crRNA2 combination was selected for further optimization. The CRISPR/Cas13a lateral flow detection could detect 1 × 101 copies/µL of DNA in the reaction when the amount of RAA amplification product was increased from 1 µL to 2 µL, which was the detection limit of this method (**Figure 3C**).

### Specificity of Cas13a lateral flow detection

**Figure 4:**
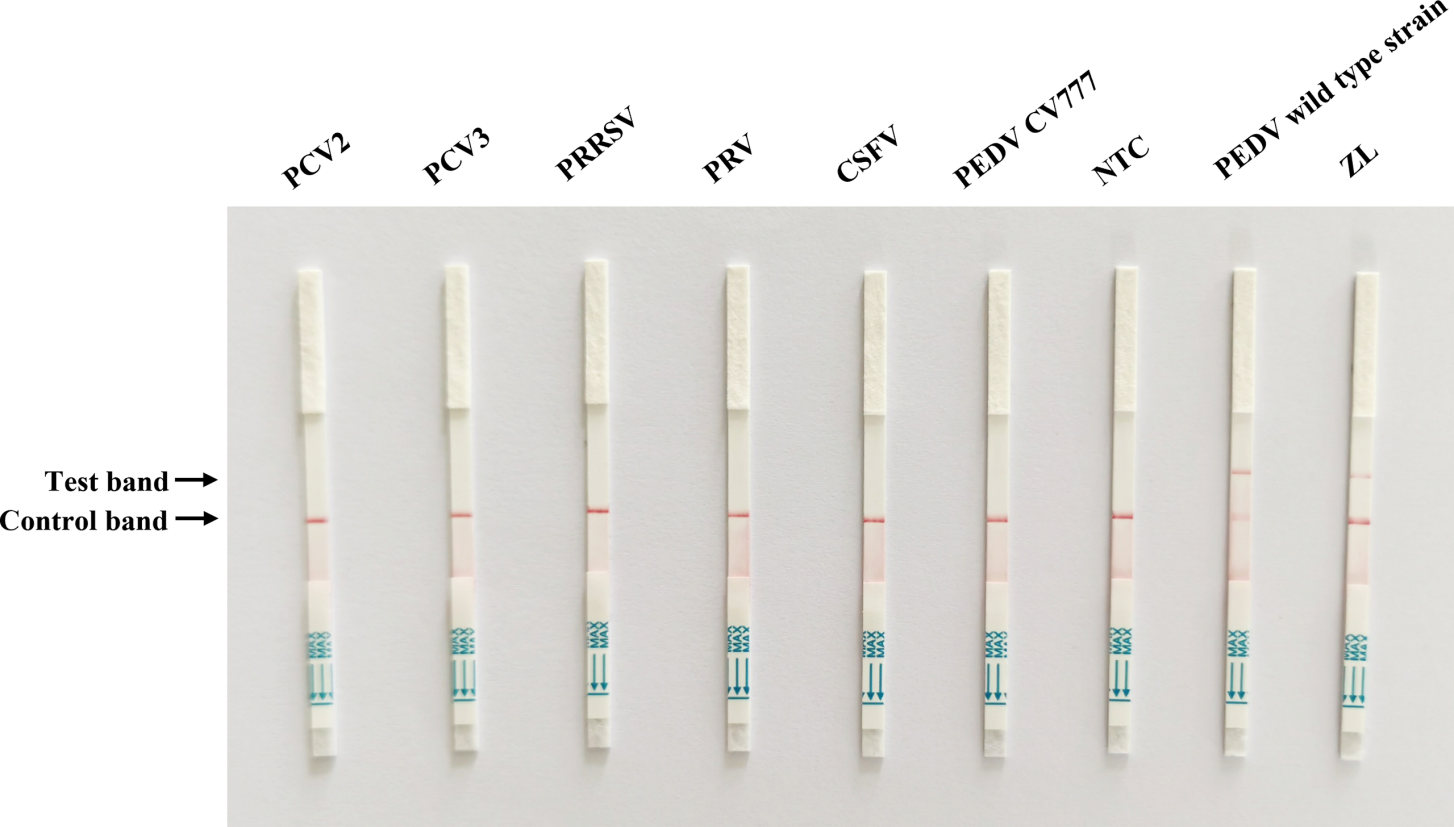
Specificity test of Cas13a lateral flow detection. Porcine circovirus type 2 (PCV2), porcine circovirus type 3 (PCV3), porcine reproductive and respiratory syndrome virus (PRRSV), pseudorabies virus (PRV), classical swine fever virus (CSFV), and PEDV attenuated vaccine strain CV777 were tested. ZL, pMD19T-ORF3 standard plasmid and NTC, negative control.

To evaluate the specificity of Cas13a lateral flow detection, the established method was used to assess the specificity detection of the PEDV wild-type and vaccine strain, as well as other porcine pathogens, including PCV2, PCV3, PRRSV, PRV, and CSFV. The primer2 + crRNA2 combination was selected for detection. The positive band was observed on the lateral flow detection strip of PEDV wild-type strain. The lateral flow detection strips of other viruses had no positive bands (**Figure 4**). The results indicated the high specificity of the method for the detection of the PEDV wild-type strain.

### Detection of clinical samples by Cas13a lateral flow detection

**Figure 5:**
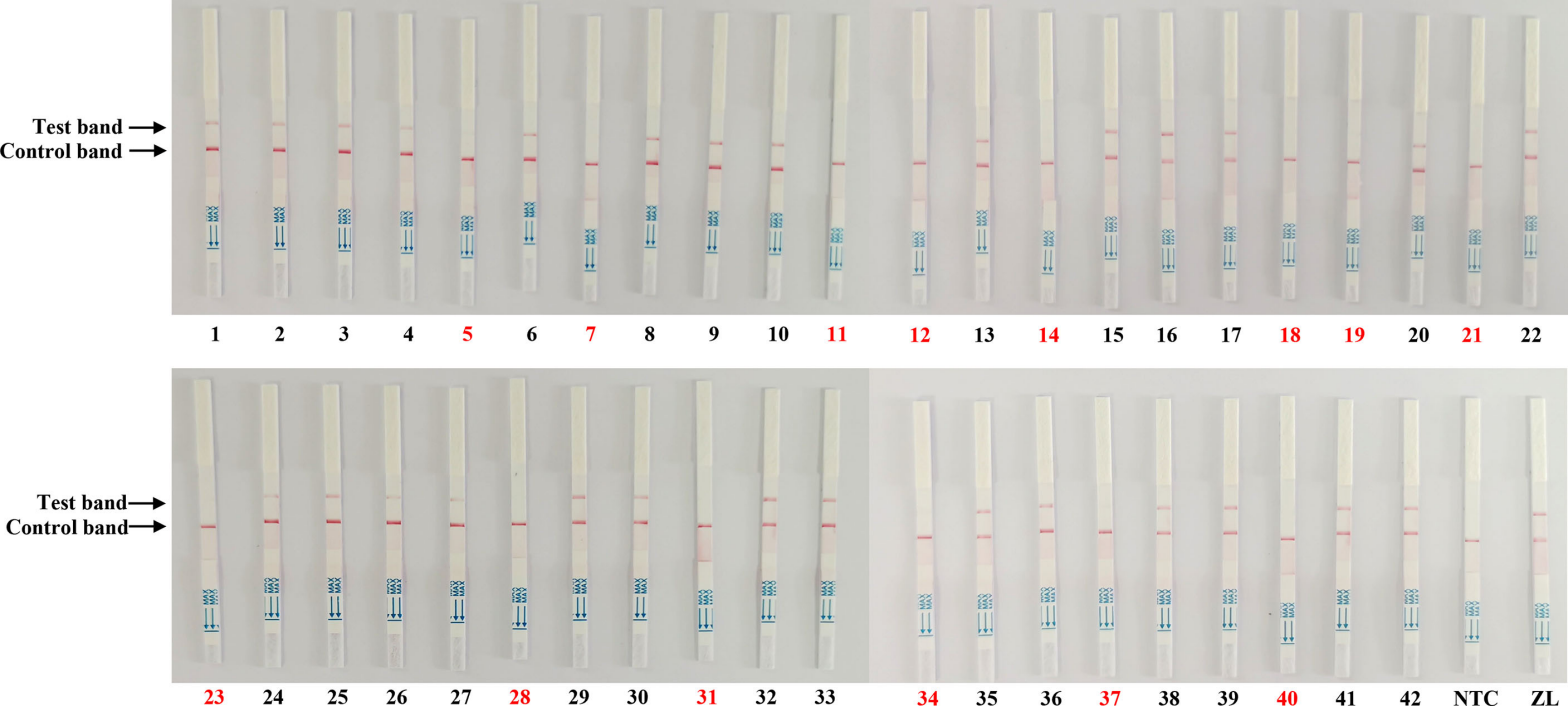
Clinical sample detection by the Cas13a lateral flow detection. The samples marked with black and red numbers are PEDV-positive and PEDV-negative samples, respectively. Forty-two samples were used for testing. ZL, pMD19T-ORF3 standard plasmid and NTC, negative control.

To validate the reliability of the Cas13a lateral flow detection in PEDV wild-type strains from vaccine strains in clinical samples, 42 qRT-PCR-tested tissue samples collected from different farms were detected by Cas13a lateral flow. As shown in **Table 2**, the qRT-PCR displayed 29 tissue samples were PEDV-positive and 13 samples were PEDV-negative, including six samples from piglets inoculated with the attenuated vaccine CV777. The Cas13a lateral flow detected 1 false-negative result obtained from the 29 detected PEDV positive samples with the coincidence rate of 96.6%, compared with qRT-PCR (**Figure 5**). The kappa value (κ) of two methods was 0.98 (**Table 2**). These data indicated that Cas13a lateral flow detection had high specificity and sensitivity in diagnostic performance for PEDV and could be used for on-site clinical PEDV detection.

**Table:** Table 2 Detection results of clinical samples in Cas13a lateral flow detection and qRT-PCR assays.

Assay	Number of samples		Comparison of two methods
	Positive	Negative	Kappa
qRT-PCR	29	13	0.98
Cas13a lateral flow detection	28	14	

## Discussion

PED is a highly contagious infectious disease that can cause diarrhea, vomiting, dehydration, and high lethality in suckling piglets. PEDV has high rates of infection and fatality in many provinces and cities in China, causing serious economic losses to the pig industry (Sun et al., 2012; Wang et al., 2016; Liang et al., 2020). During the prolonged PEDV epidemic, the virus has adapted to the environmental differences in different regions by mutating. The appearance of mutant strains has hindered the efficacy of vaccines in protecting pig herds (Zhang et al., 2021). The establishment of a differential diagnosis method that distinguishes between PEDV wild-type and vaccine strains could help to reduce the culling of immunized pigs. There are many laboratory detection methods for PEDV, but most of the detection methods require professional operation or special equipment, which are not suitable for large-scale clinical detection. Therefore, the establishment of a practical and effective PEDV pathogen detection is important in PEDV epidemiological investigations and differential diagnosis.

The CRISPR/Cas13a system can specifically cut the target single-strand RNA and has a non-specific collateral cleavage ability. These characteristics and the generation and development of the SHERLOCK technology have provided technical support and design ideas for the application of the CRISPR/Cas13a system in nucleic acid detection (Myhrvold et al., 2018). Chang et al. (2020) used CRISPR/Cas13a to detect PRRSV at 37°C with a detection limit of 172 copies/μL. No cross-reactions with PCV-2, PPV, CSFV, and PRV were evident (Chang et al., 2020). Liu et al. (2019a) designed a specific crRNA with the *in vitro* transcribed RNA of the hemagglutinin gene of H7N9 as the target, combined with RAA, and established a CRISPR/Cas13a-based rapid detection method for H7N9. Patchsung et al. (2020) reported a CRISPR/Cas13a-based diagnostic tool for rapid detection of SARS-CoV-2. This diagnostic tool produces results comparable in accuracy to conventional real-time PCR tests. It is well known that real-time PCR is considered the gold standard test for pathogenic genomic detection. However, real-time PCR must be performed in a well-equipped and professionally operated diagnostic laboratory and is not suitable for on-site detection. Additionally, diagnostic laboratories are usually located far from farms, which can lead to delayed results. The new detection method that combines CRISPR/Cas13a and lateral flow readout can overcome this shortcoming. The Cas13a lateral flow detection can be used for on-site PEDV detection with a sensitivity of 10 copies per reaction and does not require expensive instrumentation. The above further indicate the prowess of this method for the molecular detection of pathogens.

In the present study, the ORF3 gene of PEDV wild-type strain and vaccine strain were compared and analyzed. The crRNA was designed for the ORF3 deletion region to establish a clinical rapid CRISPR/Cas13a lateral flow detection method. The detection sensitivity of Cas13a lateral flow detection reached 101 copies/µL. Wang et al. (2020b) developed a real-time reverse transcription recombinase polymerase amplification assay for PEDV, with a lower limit of detection of 300 copies. The TaqMan probe-based real-time PCR technique targeting the ORF3 deletion region developed by Liu et al. (2019b) has a detection limit of 37 copies. The CRSIRR/Cas13a lateral flow detection method developed in this study is more sensitive than these two methods. The present results also showed that the method had no cross-reactivity with other swine disease viruses, and could specifically distinguish the PEDV wild-type strain from the vaccine strain. To evaluate the suitability of the technique for clinical applications, the coincidence rate of Cas13a lateral flow detection with qRT-PCR was calculated. The demonstrated reliability and availability of the Cas13a lateral flow detection method indicated its value for field diagnosis of PEDV clinical cases.

In summary, the CRISPR/Cas13a lateral flow detection was established to identify PEDV classic attenuated vaccine strains and wild-type strains. The approach had the advantages of rapid operation, high sensitivity, no limitation of laboratory equipment, and visualization of results. The method was suitable for on-site rapid detection of clinical PEDV and improved the emergency detection ability of PEDV.

## Data availability statement

The original contributions presented in the study are included in the article/Supplementary Material. Further inquiries can be directed to the corresponding authors.

## Author contributions

DY, LY, and HG were responsible for sampling and sample testing. JW and XS analyzed the data. DY and RZ wrote the paper. XP and YD edited the paper. All authors contributed to the article and approved the submitted version.

## Funding

This study was supported financially by the Talent Project of Anhui Academy of Agricultural Sciences (XJBS-202107 and 202108), Anhui Province Pig Industry Technology System (AHCYJSTX-13), the Key Research and development program of Anhui Province (202204c06020009), the Special Fund for Anhui Agriculture Research System (AHCYJXTX-05-13) and the Natural Science Foundation of Anhui (2008085MC91).

## Conflict of interest

The authors declare that the research was conducted in the absence of any commercial or financial relationships that could be construed as a potential conflict of interest.

## Publisher’s note

All claims expressed in this article are solely those of the authors and do not necessarily represent those of their affiliated organizations, or those of the publisher, the editors and the reviewers. Any product that may be evaluated in this article, or claim that may be made by its manufacturer, is not guaranteed or endorsed by the publisher.
